# Association of Medicare Mandatory Bundled Payment Program With the Receipt of Elective Hip and Knee Replacement in White, Black, and Hispanic Beneficiaries

**DOI:** 10.1001/jamanetworkopen.2021.1772

**Published:** 2021-03-22

**Authors:** Hyunjee Kim, Thomas H. A. Meath, Ana R. Quiñones, K. John McConnell, Said A. Ibrahim

**Affiliations:** 1 Center for Health Systems Effectiveness, Oregon Health & Science University, Portland; 2 Department of Family Medicine, Oregon Health & Science University, Portland; 3 Department of Healthcare Policy & Research, Weill Cornell Medicine, Cornell University, New York

## Abstract

**Importance:**

The Comprehensive Care for Joint Replacement (CJR) model was designed to reduce the cost and improve the quality of hip or knee replacement among Medicare beneficiaries. Yet whether this model may exacerbate existing racial/ethnic disparities in access to the surgery is unclear.

**Objective:**

To examine the association of the CJR model with the receipt of elective hip or knee replacement across White, Black, and Hispanic Medicare beneficiaries.

**Design, Setting, and Participants:**

Retrospective cohort study of Medicare claims from 2013 through 2017 among White, Black, and Hispanic Medicare beneficiaries undergoing elective joint replacement in 65 treatment (selected for CJR participation) and 101 control metropolitan statistical areas (MSAs).

**Exposures:**

Starting in April 2016, hospitals in the treatment MSAs were required to participate in the CJR model and were accountable for expenditures occurring during patients’ hospitalization for hip or knee replacement and 90 days after the hospital discharge.

**Main Outcomes and Measures:**

Beneficiary-level elective hip or knee replacement receipt in a given year.

**Results:**

Among 17 243 304 patients, 9 839 996 (57%) were women; 2 107 425 (12%) were age 85 years or older. Of the final sample, 14 632 434 (85%) were White beneficiaries, 1 518 629 (9%) were Black beneficiaries, and 1 092 241 (6%) were Hispanic beneficiaries. The CJR model was associated with an increase of 1.6 elective hip or knee replacements per 1000 beneficiary-years for Hispanic beneficiaries (95% CI, 0.06-2.05) and a decrease of 0.64 replacements for Black beneficiaries (95% CI, −1.25 to −0.02). No evidence was found for any changes for White beneficiaries per 1000 beneficiary-years (0.04 replacements, 95% CI, −0.35 to 0.42 replacements). The Black-White difference in the rate of elective hip or knee replacement per 1000 beneficiary-years further widened by 0.68 replacements (−0.68, 95% CI, −1.20 to −0.15).

**Conclusions and Relevance:**

In this cohort study, the CJR model was associated with increased receipt of elective hip or knee replacement among Hispanic beneficiaries, decreased receipt among Black beneficiaries, and no change in receipt among White beneficiaries. The decreased receipt of elective hip or knee replacement among Black beneficiaries may suggest that value-based payment models, including the CJR model, could be monitored for unintended consequences. However, the lack of similar findings among Hispanic beneficiaries suggests that payment models may have differential impacts across racial/ethnic groups.

## Key Points

**Question:**

Is the Medicare Comprehensive Care for Joint Replacement (CJR) model associated with the receipt of elective hip or knee replacement surgery in White, Black, and Hispanic beneficiaries?

**Findings:**

In this cohort study of 49 595 269 beneficiary-year observations from 17 243 304 patients, the CJR model was associated with increases in the receipt of elective hip or knee replacement for Hispanic beneficiaries, decreases for Black beneficiaries, and no changes for White beneficiaries.

**Meaning:**

In this study, the decrease in the receipt of hip or knee replacement among Black beneficiaries suggests a need for value-based payment models to be monitored for unintended consequences; the lack of similar findings among Hispanic beneficiaries may indicate that payment models have differential associations across racial/ethnic groups.

## Teaser

This cohort study examines the association of the Medicare Comprehensive Care for Joint Replacement model with the receipt of elective joint replacement across White, Black, and Hispanic Medicare beneficiaries.

## Introduction

Hip or knee replacement surgery (joint replacement) is an effective treatment option that can improve the quality of life for people with severe arthritis.^1^ However, the surgery has been marked by striking racial/ethnic disparities in receipt of joint replacement.^2,3^ Black and Hispanic individuals in the US are approximately 40%-50% less likely to receive elective joint replacement than Whites,^4,5,6,7,8^ a discrepancy that persists even after controlling for comorbidities and insurance status.^7,9,10,11^

The Comprehensive Care for Joint Replacement (CJR) model, Medicare’s mandatory bundled payment model, was designed to reduce health care expenditures for joint replacement and improve the quality of joint replacement care. Under the CJR model, hospitals are accountable for expenditures and the quality of care for patients receiving joint replacement during care episodes (hospitalization for the surgery and care in the 90 days after discharge). If expenditures for the episode exceed a quality-adjusted spending limit, hospitals pay a penalty. If expenditures are lower than the limit, hospitals receive a bonus.

The CJR model was implemented in April 2016 and continued through December 2020, with potential extension until 2023.^12,13^ The Centers for Medicare and Medicaid Services (CMS) randomly selected 67 metropolitan statistical areas (MSAs) for CJR implementation. Hospitals in the 67 MSAs were required to participate in the CJR model until 2017, but participation was mandatory in only 34 of the 67 MSAs beginning in 2018.^12^

The CJR model may exacerbate existing racial/ethnic disparities in receipt of elective joint replacement. The CJR model does not include risk adjustment for patients’ preexisting social or medical complexity in setting an expenditure limit for each hospital. Instead, expenditure targets are weighted means of a hospital's historical expenditure and regional historical expenditure. These historical targets may create incentives for hospitals to avoid admitting patients with socially and medically complex situations if they perceive these patients will incur higher costs during the joint replacement surgery care episode. For example, patients without reliable transportation may require a lengthy stay at a skilled nursing facility and incur higher costs than patients who go home and recover quickly. Because Black and Hispanic individuals in the US disproportionately represent socially and medically complex populations, the CJR model has the potential to widen existing racial/ethnic disparities in the receipt of elective joint replacement. We assessed the association of the CJR model with the receipt of elective joint replacement in White, Black, and Hispanic Medicare beneficiaries.

## Methods

The institutional review board at Oregon Health & Science University approved this cohort study with a waiver of informed consent because seeking informed consent from all patients included in the study was infeasible and the risk to study participants was minimal. This study followed the Strengthening the Reporting of Observational Studies in Epidemiology (STROBE) reporting guideline for cohort studies.

### Study Setting

The CMS stratified MSAs that were eligible for CJR participation into 8 groups based on population size and historical episode cost. The CMS then randomly selected MSAs for CJR participation within each of the strata, applying higher selection probabilities in the strata of higher historical cost. This approach led to 67 treatment MSAs and 104 control MSAs.^12^ We excluded the San Juan MSA from the control MSAs because it was affected by Hurricane Maria in 2017. We also excluded 2 treatment and 2 control MSAs that did not have at least 20 White, 20 Black, and 20 Hispanic Medicare beneficiaries annually. Our sample contained 65 treatment and 101 control MSAs (eMethods 1 in the Supplement).

### Sample Selection

We included all White, Black, and Hispanic Medicare beneficiaries (but not Asian/Pacific Islander beneficiaries, American Indian/Alaska Native beneficiaries, or beneficiaries with other racial/ethnic backgrounds) in our study setting between 2013 and 2017. However, we excluded observations in 2016, treating year 2016 as a washout period given the CJR model was implemented in April 2016. We excluded beneficiaries who were younger than 66 years, eligible for Medicare because of kidney failure, or were not continuously enrolled in Medicare Part A and B throughout each year (eFigure in the Supplement).

### Data

We obtained 100% each beneficiary’s demographic and health characteristics from the Medicare Master Beneficiary Summary File and 100% receipt of elective joint replacement from the Medicare inpatient claims. We used the Area Health Resources Files and CMS documentation for MSA-level characteristics.^14,15^

### Outcome

The outcome was a binary variable indicating whether each beneficiary received an elective joint replacement in a given year (eMethods 2 in the Supplement). Under the CJR model, hospitals were accountable for expenditures for patients who received joint replacement as a result of hip fracture as well as elective joint replacement. In the main analysis, however, we excluded post–hip fracture joint replacement in the outcome because hip fractures require an emergent replacement surgery and hospitals are thus limited in their ability to select hip fracture patients for joint replacement.^16^ We conducted a sensitivity analysis in which we expanded the outcome to receipt of elective or hip fracture joint replacement. We identified joint replacement due to hip fracture by using *International Classification of Diseases, Ninth Revision (ICD-9)* and *International Statistical Classification of Diseases and Related Health Problems, Tenth Revision (ICD-10)* diagnosis codes provided by the CMS.^12^

### Race/Ethnicity

We identified patient race/ethnicity using the Research Triangle Institute race field in the Master Beneficiary Summary Files. This field was created by taking the race/ethnicity code used by the Social Security Administration and applying an algorithm that identifies additional Hispanic and Asian or Pacific Islander beneficiaries based on each person’s first or last name. This field is known to be more accurate than race/ethnicity in the CMS enrollment database.^17,18^

### Statistical Analysis

We estimated a difference-in-differences-in-differences linear regression model to examine changes in the likelihood of receiving elective joint replacement under the CJR model for White, Black, and Hispanic beneficiaries.^19^ The unit of analysis was beneficiary-year. The key explanatory variables were (1) the interaction between a treatment MSA binary measure (ie, whether a person lived in one of the treatment vs control MSAs) and a post-CJR binary measure (ie, whether an observation was from the post-CJR period: year 2017 vs pre-CJR period: 2013-2015) and (2) 3-way interactions between a treatment MSA measure, a post-CJR measure, and race/ethnicity binary measures (ie, Black and Hispanic beneficiary binary measure with White beneficiary as a reference group). The coefficient of the first interaction term measured changes under the CJR model for White beneficiaries. Because the coefficient of 3-way interaction terms measured changes in Black-White and Hispanic-White differences under the CJR model, we calculated the changes under the CJR model for Black and Hispanic beneficiaries by summing the coefficients of the first interaction term and those of the 3-way interactions.

The model also included interaction terms between race/ethnicity and post-CJR binary measure, interaction terms between race/ethnicity and treatment MSA measure, race/ethnicity measures, measures of each MSA, and measures of each year. Models did not adjust for the main effect of treatment MSA or post-CJR measure because they were perfectly collinear with MSA and year measures, respectively. We also adjusted for beneficiary age groups, sex, receipt of joint replacement in the preceding year, and 65 chronic health conditions as well as MSA characteristics. The explanatory variables are explained in eMethods 2 and the model in eMethods 3 in the Supplement.

We clustered standard errors at the MSA level to account for correlation across multiple observations from the same MSAs and beneficiaries and applied sampling weights to account for oversampling during treatment MSA selection (eMethods 1 in the Supplement).^20^ All statistical tests were 2-sided, and statistical significance was set at *P* < .05. Analyses were conducted using Stata, version 16. (StataCorp LLC).

We conducted 3 secondary analyses. First, we used combined measures for race/ethnicity and dual Medicare/Medicaid beneficiary status instead of a race/ethnicity measure and assessed changes in outcomes under the CJR model across White beneficiaries without Medicaid, White beneficiaries with Medicaid, Black beneficiaries without Medicaid, Black beneficiaries with Medicaid, Hispanic beneficiaries without Medicaid, and Hispanic beneficiaries with Medicaid. Although Black and Hispanic beneficiaries are disproportionately represented among beneficiaries with low socioeconomic status, there is significant heterogeneity in socioeconomic status within each racial/ethnic group. This analysis allowed us to assess whether observed changes in the main analysis were concentrated within low socioeconomic status groups within each racial/ethnic group.

Second, we performed an intention-to-treat analysis based on the approach used in prior studies.^21,22^ In the intention-to-treat analysis, we included 74 treatment and 103 control MSAs that were initially eligible for CJR participation before the CMS revised the CJR eligibility criteria in November 2015 (eMethods 1 in the Supplement). Third, we split the outcome into the receipt of elective hip replacement and the receipt of elective knee replacement. We then repeated the same analysis.

## Results

The final sample consisted of 49 595 269 beneficiary-year observations from 17 243 304 unique beneficiaries (9 839 996 [57%] women; 2 107 425 [12%] age ≥85 years; 14 632 434 [85%] White beneficiaries, 1 518 629 [9%] Black beneficiaries, and 1 092 241 [6%] Hispanic beneficiaries).

Table 1 presents White, Black, and Hispanic beneficiary characteristics during the pre-CJR period. Black and Hispanic beneficiaries were younger than White beneficiaries (30.4% of White beneficiaries were 70 years old or younger compared with 35.1% of Black beneficiaries and 34.5% of Hispanic beneficiaries), more likely to have Medicaid (6.1% of White beneficiaries compared with 21.7% of Black beneficiaries and 37.4% of Hispanic beneficiaries), and more likely to have chronic conditions, including obesity (9.4% of White beneficiaries compared with 14.0% of Black beneficiaries and 11.5% of Hispanic beneficiaries) and diabetes (25.7% of White beneficiaries compared with 42.4% of Black beneficiaries and 40.9% of Hispanic beneficiaries).

**Table 1.** Unadjusted White, Black, and Hispanic Medicare Beneficiary Characteristics During Pre-Comprehensive Care for Joint Replacement (CJR) Period, 2013-2015

Characteristic	White	Black	Hispanic
	Weighted, No. (%)	Weighted, No. (%)	Weighted, No. (%)
Weighted No.	31 866 365	3 060 569	2 071 195
Female	18 331 688 (57.5)	1 855 690 (60.6)	1 182 176 (57.1)
Medicaid-enrolled	1 943 017 (6.1)	662 846 (21.7)	774 499 (37.4)
Age, y			
66-70	9 679 277 (30.4)	1 073 104 (35.1)	714 907 (34.5)
71-75	7 638 166 (24.0)	769 204 (25.1)	524 028 (25.3)
76-80	5 772 123 (18.1)	546 992 (17.9)	377 524 (18.2)
81-85	4 463 646 (14.0)	353 536 (11.6)	255 755 (12.3)
86-90	2 895 698 (9.1)	206 522 (6.7)	138 877 (6.7)
91-95	1 147 968 (3.6)	83 734 (2.7)	48 563 (2.3)
≥96	269 487 (0.8)	27 477 (0.9)	11 541 (0.6)
Chronic condition			
Hypertension	19 678 431 (61.8)	2 237 669 (73.1)	1 286 290 (62.1)
Rheumatoid arthritis/osteoarthritis	10 895 937 (34.2)	1 084 636 (35.4)	685 378 (33.1)
Diabetes	8 198 464 (25.7)	1 296 538 (42.4)	847 717 (40.9)
Chronic kidney disease	5 523 839 (17.3)	801 667 (26.2)	392 266 (18.9)
Congestive heart failure	4 535 170 (14.2)	594 109 (19.4)	310 012 (15.0)
Obesity	3 004 762 (9.4)	427 528 (14.0)	237 601 (11.5)
Osteoporosis	2 540 651 (8.0)	138 459 (4.5)	177 569 (8.6)

The Figure depicts the unadjusted rate of elective joint replacements per 1000 White, Black, and Hispanic beneficiaries across treatment and control MSAs between 2013 and 2017. White beneficiaries were consistently more likely to receive elective joint replacement than Black and Hispanic beneficiaries. For example, approximately 13.2 of 1000 White beneficiaries in treatment MSAs received elective joint replacement in 2013 compared with 7.7 for Black beneficiaries and 7.3 for Hispanic beneficiaries.

**Figure.**
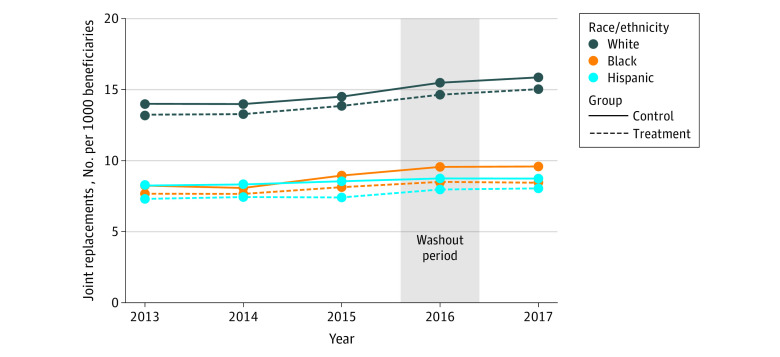
Unadjusted Rate of Elective Joint Replacements per 1000 White, Black, and Hispanic Beneficiaries Across Treatment and Control Metropolitan Statistical Areas (MSAs) Between 2013 and 2017 (N = 49 595 269) Joint replacement rate is unadjusted and does not account for the stratified random sampling method or potential confounding explanatory variables.

The Figure depicts unadjusted changes in the rates of elective joint replacements across White, Black, and Hispanic beneficiaries. The joint replacement rate for White beneficiaries in treatment MSAs increased in parallel with the rate in control MSAs after CJR implementation (rates in treatment MSAs increased by 1.2 from 2015 to 2017, while rates in control MSAs increased by 1.4), indicating no association between the CJR model and changes in White beneficiaries’ joint replacement rate. Black beneficiaries in treatment MSAs experienced increases in the rate of elective joint replacements in 2017, but these increases were less than those in control MSAs (rates for Black beneficiaries in treatment MSAs increased by 0.3 from 2015 to 2017 compared with an increase of 0.6 in control MSAs). This finding suggests that the CJR model may have affected the rate of elective joint replacements among Black beneficiaries. For Hispanic beneficiaries, the rate of joint replacements in control MSAs was consistent throughout the study period, but the rate in treatment MSAs slightly increased in 2017 (rates for Hispanic beneficiaries in control MSAs increased by 0.6 in treatment MSAs from 2015 to 2017 compared with an increase of 0.2 in control MSAs). This finding suggests a potential association of the CJR model with a relative increase in the rate of joint replacements among Hispanic beneficiaries.

Table 2 presents the association of the CJR model with changes in the rate of elective joint replacements per 1000 beneficiary-years across White, Black, and Hispanic beneficiaries. The rate of joint replacements increased for Hispanic beneficiaries by 1.06 replacements per 1000 beneficiary-years (95% CI, 0.06-2.05; 14.3% increase from the pre-CJR rate in treatment MSAs). In contrast, we observed a decrease of 0.64 replacements per 1000 Black beneficiary-years (95% CI, −1.25 to −0.02; an 8.2% reduction from the pre-CJR rate in treatment MSAs). We found no evidence for any changes in the rate of elective joint replacements per 1000 White beneficiary-years (0.04 replacements; 95% CI, −0.35 to 0.42). Taken together, these changes suggest a widening of Black-White disparities in receipt of joint replacement by 0.68 replacements (95% CI, −1.20 to −0.15) per 1000 beneficiary-years under the CJR model (Table 3). For additional context, Table 3 also reports the unadjusted pre-CJR difference in rates per 1000 person-years for Black beneficiaries compared to White beneficiaries and Hispanic beneficiaries compared to White beneficiaries. We found no evidence for changes in Hispanic-White disparities in the receipt of joint replacements (1.02 replacements; 95% CI, −0.03 to 2.07).

**Table 2.** Change in the Rate of Elective Joint Replacements per 1000 White, Black, and Hispanic Beneficiary-Years Under the CJR Model (N = 49 595 269)

Race	MSAs						Treatment vs Control	
	Treatment			Control				
	CJR period^a^		Difference^a^	CJR period^a^		Difference^a^	Change associated with the CJR model (95% CI)^b^	*P* value
	Pre (2013-2015)	Post (2017)		Pre (2013-2015)	Post (2017)			
White	13.46	15.03	1.58	14.17	15.86	1.70	0.04 (−0.35 to 0.42)	.85
Black	7.82	8.45	0.63	8.42	9.59	1.17	−0.64 (−1.25 to −0.02)	.04
Hispanic	7.38	8.05	0.66	8.37	8.74	0.36	1.06 (0.06 to 2.06)	.04

Abbreviations: CJR, Comprehensive Care for Joint Replacement; MSAs, metropolitan statistical areas.

^a^ Unadjusted estimates.

^b^ Adjusted estimates indicate changes in the receipt of joint replacement associated with CJR for White, Black, and Hispanic beneficiaries.

**Table 3.** Change in Black-White and Hispanic-White Disparities in the Rate of Elective Joint Replacements Under the CJR Model (N = 49 595 269)

Disparity	Disparities in treatment MSAs pre-CJR^a^	Changes in disparities associated with the CJR model (95% CI)^b^	*P* value
Black-White	−5.63	−0.68 (−1.20 to −0.15)	.01
Hispanic-White	−6.08	1.02 (−0.03 to 2.07)	.06

Abbreviations: CJR, Comprehensive Care for Joint Replacement; MSAs, metropolitan statistical areas.

^a^ Unadjusted Black-White and Hispanic-White disparities in the receipt of joint replacement in the treatment MSAs during the pre-CJR period (2013-2015). Black-White disparities are calculated by subtracting the rate of joint replacement per 1000 White beneficiary-years from the rate of joint replacement per 1000 Black beneficiary-years. For example, −5.63 for Black-White disparities indicates that the rate of joint replacement for 1000 Black beneficiary-years was lowered by 5.63 replacements than the corresponding rate.

^b^ Adjusted changes in Black-White and Hispanic-White disparities in the receipt of joint replacement associated with the CJR model (ie, changes in joint replacement rates for Black beneficiaries minus changes in joint replacement rates for White beneficiaries; changes in joint replacement rates for Hispanic beneficiaries minus changes in joint replacement rates for White beneficiaries). For example, −0.68 for Black-White disparities indicates that the disparity in the rate of joint replacement between Black and White beneficiaries increased by 0.68 replacements.

Table 4 presents changes in the rate of elective joint replacements under the CJR model across White, Black, and Hispanic beneficiaries combined with their Medicaid coverage status. Among Hispanic beneficiaries, the CJR model was associated with an increased rate of joint replacements among those with Medicaid (increase of 2.04 replacements; 95% CI, 0.45-3.62). We found no evidence for any changes in the other groups.

**Table 4.** Change in the Rate of Elective Joint Replacements per 1000 White, Black, and Hispanic Beneficiary-Years With and Without Medicaid Coverage Under the CJR Model (N = 49 595 269)

Variable	Treatment MSAs			Control MSAs			Treatment vs Control	
	CJR^a^		Difference^a^	CJR^a^		Difference^a^	Change associated with the CJR model (95% CI)^b^	*P* value
	Pre (2013-2015)	Post (2017)		Pre (2013-2015)	Post (2017)			
**White beneficiaries**								
Without Medicaid	14.03	15.67	1.64	14.62	16.33	1.71	0.05 (−0.35 to 0.45)	.80
With Medicaid	5.68	5.73	0.05	6.30	6.83	0.52	−0.04 (−0.71 to 0.63)	.91
**Black beneficiaries**								
Without Medicaid	8.66	9.45	0.80	9.09	10.24	1.15	−0.55 (−1.23 to 0.12)	.11
With Medicaid	5.17	4.90	−0.27	5.71	6.56	0.85	−0.76 (−1.53 to 0.02)	.05
**Hispanic beneficiaries**								
Without Medicaid	7.87	8.72	0.85	8.45	9.18	0.73	0.45 (−0.28 to 1.18)	.23
With Medicaid	6.74	7.01	0.27	8.21	7.61	−0.60	2.04 (0.45 to 3.62)	.01

Abbreviations: CJR, Comprehensive Care for Joint Replacement; MSAs, metropolitan statistical areas.

^a^ Unadjusted estimates.

^b^ Adjusted estimates.

The intention-to-treat analysis results (eTable 2 in the Supplement) were similar: the rate of elective joint replacements increased for Hispanic beneficiaries (1.02 replacements; 95% CI, 0.02-2.01) but decreased for Black beneficiaries (−0.7 replacements; 95% CI, −1.31 to −0.09). In the sensitivity analysis that expanded the outcome to the receipt of elective or fracture replacement, we found no evidence of any changes across White, Black, and Hispanic beneficiaries (eTable 3 in the Supplement). The lack of changes may reflect hospitals’ inability to select patients with an emergent, as opposed to elective, medical need. In a separate sensitivity analysis, we split the outcome into any receipt of elective hip replacement and any receipt of elective knee replacement and found that the CJR model was associated with a relative decrease in the rate of elective knee replacements among Black beneficiaries and a relative increase in the rate of elective knee replacements among Hispanic beneficiaries (eTable 4 in the Supplement). However, we found no evidence for changes in the rate of elective hip replacements among both Black and Hispanic beneficiaries (eTable 5 in the Supplement). We found no significant differences in pre-CJR trends in the outcome between treatment and control MSAs across racial/ethnic groups (eTable 1 in the Supplement), supporting the parallel pre-trends assumption.

## Discussion

Previous studies have shown that US Black and Hispanic individuals were 40% to 50% less likely to receive elective joint replacements than US White individuals, but most of these studies used data from before 2010.^4,5,6,7^ Using a more recent, comprehensive sample of 49.6 million Medicare beneficiaries, we found persistent racial/ethnic disparities in receiving elective joint replacement, with White beneficiaries receiving elective joint replacement almost twice as often as Black or Hispanic beneficiaries. Our finding indicates that continued efforts to improve the Black-White and Hispanic-White disparities in the receipt of elective joint replacements are warranted.

We found the increase in the rate of elective joint replacements among Black beneficiaries in treatment MSAs after CJR implementation to be smaller than that in control MSAs, indicating that the CJR model may have been associated with a relative decrease in the rates of joint replacements among Black beneficiaries. In contrast, CJR was associated with a relative increase and no changes in the rate of joint replacements among Hispanic and White beneficiaries, respectively. The size of absolute changes among Black and Hispanic beneficiaries (reduction of 0.64 replacements per 1000 Black beneficiary-years and increase of 1.06 replacements per 1000 Hispanic beneficiary-years) was small. However, the relative changes were more substantial, representing an 8.2% reduction from the pre-CJR rate among Black beneficiaries in treatment MSAs and a 14.3% increase from the pre-CJR rate among Hispanic beneficiaries in treatment MSAs. These changes further widened Black-White disparities in the receipt of joint replacements, exacerbating long-standing Black-White disparities. We found no evidence for any changes in Hispanic-White disparities associated with the CJR model, but the rate of joint replacements remained more than twice as high for White beneficiaries than for Hispanic beneficiaries.

We hypothesized that hospitals might limit admissions of patients with socially and medically complex situations for elective joint replacement to avoid financial penalties under the CJR model. Our finding of decreases in the receipt of elective joint replacement among Black beneficiaries is consistent with this hypothesis because Black beneficiaries are more likely to have social and medical complexity than White beneficiaries. However, we did not observe similar patterns among Hispanic beneficiaries. The CJR model was associated with increases in the receipt of elective joint replacement among Hispanic beneficiaries, primarily among Hispanic beneficiaries with Medicaid. This finding was unexpected because a disproportionately high proportion of Hispanic beneficiaries, particularly those with Medicaid coverage, represent populations with socially and medically complex situations. A variety of factors, including varying degrees of local context and culture, structural racism, and complex socioeconomic factors (eg, presence of social support for beneficiaries, access to reliable transportation, and home environment amenable to rehabilitation after the surgery), may be factors in how changes in payment systems differentially affect racial/ethnic groups.

Furthermore, changes in payment systems may have differing consequences for patients at various points of care. Although this study found that the CJR model was associated with decreases in receipt of elective joint replacement among Black beneficiaries, a previous study^23^ reported that the CJR model was associated with decreases in hospital readmissions for Black joint replacement patients despite their reduced use of institutional post-acute care, a care setting that provides more intensive rehabilitation care but is more expensive. Thus, the implications of the CJR model for Black beneficiaries are nuanced. Moving away from the CJR model back to the fee-for-service payment system may improve their access to joint replacement but at the expense of lower quality of care and higher costs. An improved policy may include refinements of the CJR model, with the access to elective joint replacement an important metric to monitor.

Changes in the receipt of elective knee replacements as opposed to elective hip replacements were the primary factor in our results. The CJR model was associated with a relative decrease in the rate of elective knee replacements among Black beneficiaries and a relative increase in the rate of elective knee replacements among Hispanic beneficiaries. However, there was no evidence of significant changes in the rate of elective hip replacements among both Black and Hispanic beneficiaries. Hospitals may have found it easier to select patients for elective knee replacement due to a higher volume of elective knee replacements. More research is needed to understand why these changes were confined to elective knee replacements.

### Limitations

This study has limitations. First, the identification of Hispanic beneficiaries in Medicare Master Beneficiary Summary File was less accurate than self-reported measures, but self-reported race/ethnicity measures were not available in our data source.^24^ Misclassification of Hispanic beneficiaries into non-Hispanic White beneficiaries could wash out differences between the 2 groups, biasing our estimates of changes in the Hispanic-White disparities toward the null. Second, we used all Medicare beneficiaries as our denominator to measure the rate of joint replacements. The ideal approach would be to use Medicare beneficiaries who had clinical indications for joint replacement as a denominator, but identifying this group accurately was infeasible within our data set. To address this limitation, we accounted for a rich set of chronic conditions, including osteoarthritis and obesity, in our analyses. Third, our study only included 1 full year of post-CJR data, and thus we were unable to assess the association of the CJR model with long-term changes in the joint replacement rate across White, Black, and Hispanic beneficiaries. Fourth, our sample excluded beneficiaries aged 65 or younger with disabilities, although they were covered by the CJR model. In addition, our analyses excluded Asian/Pacific Islander individuals, Native American/Alaska Native individuals, and beneficiaries with other racial/ethnic backgrounds. Their group size was relatively small.

## Conclusions

In this study, the CJR model was associated with increases in the receipt of elective joint replacement among Hispanic beneficiaries, decreases among Black beneficiaries, and no changes among White beneficiaries compared with corresponding racial/ethnic groups in control MSAs. The decrease in the receipt of elective joint replacement among Black beneficiaries suggests that value-based payment models, including the CJR model, should be monitored for unintended consequences. However, the lack of similar findings among Hispanic beneficiaries indicates that payment models may have differential consequences for racial/ethnic groups.

## Notes

Supplement. **eMethods 1.** The Study Setting Selection and Calculation of Sampling Weights **eMethods 2.** The Outcome and Explanatory Variables **eMethods 3.** The Analytic Model **eFigure.** The Sample Selection Criteria **eTable 1.** The Parallel Pre-trends Assumption **eTable 2.** Intention to Treat Analysis: the Change in the Rate of Joint Replacements per 1,000 White, Black, and Hispanic Beneficiary-years Under the Comprehensive Care for Joint Replacement Model (N= 51,588,590) **eTable 3.** Change in the Rate of Elective or Hip Fracture Replacement per 1,000 White, Black, and Hispanic Beneficiary-year Under the Comprehensive Care for Joint Replacement Model (N=49,595,269) **eTable 4.** Change in the Rate of Elective Knee Replacement per 1,000 White, Black, and Hispanic Beneficiary-year Under the Comprehensive Care for Joint Replacement Model (N=49,595,269) **eTable 5.** Change in the Rate of Elective Hip Replacement per 1,000 White, Black, and Hispanic Beneficiary-year Under the Comprehensive Care for Joint Replacement Model (N=49,595,269)
