# Supplementary material for: Association of Medicare Mandatory Bundled Payment Program With the Receipt of Elective Hip and Knee Replacement in White, Black, and Hispanic Beneficiaries
Source: JAMA Netw Open. 2021 Mar 22;4(3):e211772. doi: 10.1001/jamanetworkopen.2021.1772 (PMC7985721; doi:10.1001/jamanetworkopen.2021.1772)
Supplement: Supplement. — eMethods 1. The Study Setting Selection and Calculation of Sampling Weights eMethods 2. The Outcome and Explanatory Variables eMethods 3. The Analytic Model eFigure. The Sample Selection Criteria eTable 1. The Parallel Pre-trends Assumption eTable 2. Intention to Treat Analysis: the Change in the Rate of Joint Replacements per 1,000 White, Black, and Hispanic Beneficiary-years Under the Comprehensive Care for Joint Replacement Model (N= 51,588,590) eTable 3. Change in the Rate of Elective or Hip Fracture Replacement per 1,000 White, Black, and Hispanic Beneficiary-year Under the Comprehensive Care for Joint Replacement Model (N=49,595,269) eTable 4. Change in the Rate of Elective Knee Replacement per 1,000 White, Black, and Hispanic Beneficiary-year Under the Comprehensive Care for Joint Replacement Model (N=49,595,269) eTable 5. Change in the Rate of Elective Hip Replacement per 1,000 White, Black, and Hispanic Beneficiary-year Under the Comprehensive Care for Joint Replacement Model (N=49,595,269) [file jamanetwopen-e211772-s001.pdf]

Supplemental Online Content

Kim H, Meath THA, Quiñones AR, McConnell KJ, Ibrahim SA. Association of Medicare mandatory bundled payment program with the receipt of elective hip and knee replacement in White, Black, and Hispanic beneficiaries. *JAMA Netw Open*. 2021;4(3):e211772. doi:10.1001/jamanetworkopen.2021.1772

eMethods 1. The Study Setting Selection and Calculation of Sampling Weights

eMethods 2. The Outcome and Explanatory Variables

eMethods 3. The Analytic Model

eFigure. The Sample Selection Criteria

eTable 1. The Parallel Pre-trends Assumption

eTable 2. Intention to Treat Analysis: the Change in the Rate of Joint Replacements per 1,000 White, Black, and Hispanic Beneficiary-years Under the Comprehensive Care for Joint Replacement Model (N= 51,588,590)

eTable 3. Change in the Rate of Elective or Hip Fracture Replacement per 1,000 White, Black, and Hispanic Beneficiary-year Under the Comprehensive Care for Joint Replacement Model (N=49,595,269)

eTable 4. Change in the Rate of Elective Knee Replacement per 1,000 White, Black, and Hispanic Beneficiary-year Under the Comprehensive Care for Joint Replacement Model (N=49,595,269)

eTable 5. Change in the Rate of Elective Hip Replacement per 1,000 White, Black, and Hispanic Beneficiary-year Under the Comprehensive Care for Joint Replacement Model (N=49,595,269)

This supplemental material has been provided by the authors to give readers additional information about their work.

© 2021 Kim H et al. *JAMA Network Open.*

eMethods 1. The study setting selection and calculation of sampling weights

Study Setting

Our study setting was determined by CMS's selection of MSAs for participation in the CJR program. There are a total of 388 MSAs in the United States. CMS selected 67 CJR MSAs through the following process:

1. CMS first excluded 192 of 388 MSAs with fewer than 400 hip/knee joint replacements per year and MSAs where bundled payments under the "Bundled Payments for Care Improvement (BPCI) Initiative" paid for more than 50% of hip/knee joint replacements. A total of 196 MSAs were eligible for CJR participation.
2. CMS then divided the remaining MSAs into four groups based on historic hip/knee joint replacements costs (i.e., costs incurred during the index hospitalization and 90 days following the hospital discharge).
3. Within each of these four groups, CMS randomly selected 75 of 196 MSAs for CJR participation, with higher probabilities of selection applied for higher-cost groups. Accordingly, 75 and 121 MSAs were treatment and control group, respectively, at this stage. Our intention-to-treat analysis included these MSAs.
4. In November 2015, CMS revised CJR eligibility criteria based on BPCI participation from July 2015-September 2015. As a result, 8 and 17 additional MSAs were excluded from the treatment and control group, respectively.
5. Among the remaining 67 and 104 treatment and control MSAs, we dropped the San Juan MSA from the control group because it was hit hard by Hurricane Maria in 2017. We also dropped 2 treatment and 2 control MSAs which did not have at least 20 White, 20 Black, and 20 Hispanic Medicare beneficiaries annually throughout our study period.
6. Our final sample included 65 treatment and 101 control MSAs.

Sampling weight

We calculated sampling weights and applied them in the main analysis to account for CJR's stratified clustered sampling approach. Based on the MSA eligibility criteria specified above, we defined the conditional probability of an MSA being selected for CJR, given that eligibility criteria are fulfilled, as:

$$P(CJR_{msa} = 1 \mid Eligibility_{msa} = 1) = \frac{Number\ of\ MSAs\ Selected\ after\ exclusion}{Number\ of\ MSAs\ Eligible\ after\ exclusion}$$

We then calculated raw weights from these probabilities using the following equation:

$$w = \begin{cases} 1/p & if\ selected = 1 \\ 1/(1-p) & if\ selected = 0 \end{cases}$$

Finally, we normalized the selection weights for each strata by dividing them by the sum of the raw strata-level selection weights, and normalized the non-selection weights by dividing them by the sum of the raw-strata-level non-selection weights.

Table 1. Summary of selection proportion for each sampling stratum

				Treatment Group		Control Group	
CJR Sampling Stratum	Population Size	Historic Cost Group	# Eligible MSAs	# CJR MSAs	Proportion of MSAs selected	# Control MSAs	Proportion of MSAs not selected
1	Below 430K	1 (Lowest)	22	7	32%	15	68%
2	Below 430K	2	18	6	33%	12	67%
3	Below 430K	3	19	8	42%	11	58%
4	Below 430K	4 (Highest)	22	11	50%	11	50%
5	Above 430K	1 (Lowest)	14	5	36%	9	64%
6	Above 430K	2	28	10	36%	18	64%
7	Above 430K	3	22	9	41%	13	59%
8	Above 430K	4 (Highest)	21	9	43%	12	57%
TOTAL	-	-	166	65	39%	101	61%

© 2021 Kim H et al. *JAMA Network Open.*

Table 2. Normalized selection and non-selection weights for each sampling stratum

			Treatment Group			Control Group		
CJR Sampling Stratum	Population Size	Historic Cost Group	Proportion of MSAs selected	Raw Sampling Weight	Normalized Sampling Weight	Proportion of MSAs not Selected	Raw Sampling Weight	Normalized Sampling Weight
1	Below 430K	1 (Lowest)	32%	3.14	0.15	68%	1.46	0.11
2	Below 430K	2	33%	3.00	0.14	67%	1.50	0.11
3	Below 430K	3	42%	2.38	0.11	58%	1.73	0.13
4	Below 430K	4 (Highest)	50%	2.00	0.10	50%	2.00	0.15
5	Above 430K	1 (Lowest)	36%	2.80	0.13	64%	1.56	0.12
6	Above 430K	2	36%	2.80	0.13	64%	1.56	0.12
7	Above 430K	3	41%	2.44	0.12	59%	1.69	0.13
8	Above 430K	4 (Highest)	43%	2.33	0.11	57%	1.75	0.13
TOTAL	-	-	-	20.91	1.00	-	13.30	1.00

© 2021 Kim H et al. *JAMA Network Open.*

eMethods 2. The outcome and explanatory variables

Outcome

The outcome was whether each beneficiary received an elective hip replacement or elective knee replacement each year during the study period. Elective hip or knee replacement surgeries were identified using a combination of the following medical classification codes[1,2] :

1) Medicare Severity Diagnosis Related Groups (MS-DRG)
    a. 469 – Major joint replacement or reattachment of lower extremity with major complication or comorbidity
    b. 470 – Major joint replacement or reattachment of lower extremity without major complication or comorbidity
2) International Classification of Diseases, Ninth Revision, Clinical Modification (ICD-9-CM) procedure codes
    a. 81.51 – Total hip replacement
    b. 81.52 – Partial hip replacement
    c. 81.54 – Total knee replacement
3) ICD-10-Procedure Coding System (ICD-10-PCS) procedure codes
    a. 0SRA* - Hip joint, Acetabular surface, right
    b. 0SRB* - Hip joint, left
    c. 0SRC* - Knee joint, right
    d. 0SRD* - Knee joint, left
    e. 0SRE* - Hip joint, Acetabular surface, left
    f. 0SRR* - Hip joint, Femoral surface, right
    g. 0SRS* - Hip joint, Femoral surface, left
    h. 0SRT* - Knee joint, Femoral surface, right
    i. 0SRU* - Knee joint, Femoral surface left
    j. 0SRV* - Knee joint, Tibial surface, right
    k. 0SRW* - Knee joint, Tibial surface, left
    l. 0SR9* - Hip joint, right
4) We excluded non-elective hip replacement surgeries using a combination of ICD-9 and ICD-10 codes provided by CMS.[3]

Explanatory variables

The three main explanatory variables were created using the Medicare Master Beneficiary Summary File and included

- Treatment indicator (if a beneficiary resided in a MSA that participated in CJR, value is 1. Otherwise, value is 0)
- Post-CJR indicator (if an observation occurred in 2017, value is 1. Otherwise, value is 0),
- Beneficiary race/ethnicity (defined using the variable rti_race_cd from the Medicare Master Beneficiary Summary File Base)
    - White: rti_race_cd = 1
    - Black: rti_race_cd = 2
    - Hispanic: rti_race_cd = 5

We also adjusted for beneficiary characteristics that previous studies have associated with increased joint replacement spending, readmissions, and complications.[4–10] These characteristics could therefore influence providers' decision to perform joint replacements for any given patient. We obtained data for beneficiary-level characteristics from the Medicare Master Beneficiary Summary File and Medicare inpatient claims. Beneficiary-level covariates included:

- Age (in 5-year intervals, one in the beginning of each year)
- Gender (female or male)
- Chronic health conditions
    - Coded as binary variables for each calendar year and measured at the end of the preceding year (e.g., presence of condition in 2013 is measured as of the end of 2012)

- We include the 27 conditions listed in the Medicare Master Beneficiary Summary Chronic Conditions file and the 40 conditions listed in the Medicare Master Beneficiary Summary File Other Chronic Conditions file for a total of 67 conditions. Examples of important conditions to adjust for include:
  - Obesity
  - Osteoarthritis
  - Chronic kidney disease
  - Diabetes
  - Opioid use disorder
  - Congestive heart failure
  - Alzheimer's/other neurological disease
  - Chronic obstructive pulmonary disease
  - Tobacco use
  - Cancer (Endometrial, breast, lung, colorectal, and prostate)

- The receipt of joint replacement(s) in the previous year

Additionally, we included the following MSA-level characteristics that could influence providers' decision to admit any given patient for joint replacement:

From the Area Health Resources File:

- # of Orthopedic surgeons measured per 100,000 residents (available for 2010, 2015, and 2016)
- # of Home health agencies measured per 100,000 residents (available for 2010-2017)
- # of Skilled nursing home beds measured per 100,000 residents (available for 2010-2017)
- # of Inpatient rehabilitation beds measured per 100,000 residents (available for 2016)
- Medicare Advantage penetration rates (2010-2017)

We calculated the Herfindahl-Hirschman Index using Medicare inpatient claims:

- Market concentration for Medicare fee-for-service joint replacements, measured by Herfindahl-Hirschman Index (HHI) – We calculate HHI for all MSAs such that, for every MSA m with n number of hospitals h:

$$HHI_m = \sum_{h=1}^{n} \left(\frac{\#\ of\ LEJRs\ performed\ in\ hospital\ h}{\#\ of\ LEJRs\ performed\ in\ MSA\ m} \right)^2 \times 10{,}000$$

From the CMS website,[11]

- BPCI penetration rates – Calculated as the proportion of elective hip/knee replacement surgeries performed at BPCI-participating hospitals out of all hip/knee replacements per year

We imputed missing data for MSA-level characteristics using a last measure carried forward approach.

eMethods 3. The analytic model

We used a difference-in-difference-in-differences ("triple-difference") approach for our main analyses. The unit of analysis was each beneficiary-year. We modeled the probability of a Medicare beneficiary i residing in MSA m during year t receiving a joint replacement using the following linear probability model:

$$Y_{imt} = \beta_0 + \beta_1(CJR_m \times Post_t \times Black_{im}) + \beta_2(CJR_m \times Post_t \times Hispanic_{im}) + \beta_3(CJR_m \times Post_t) + XB + \sum_m \phi_m MSA_m + \sum_t \gamma_t Year_t + \beta_4(CJR_m \times Black_{im}) + \beta_5(Post_t \times Black_{im}) + \beta_6(CJR_m \times Hispanic_{im}) + \beta_7(Post_t \times Hispanic_{im}) + \epsilon_{imt} \quad (1)$$

where Y_{imt} was a binary outcome that indicates whether a beneficiary i residing in MSA m received an elective joint replacement in year t. CJR_m took a value of 1 if a beneficiary resided in a treatment MSA and 0 otherwise. $Post_t$ took a value of 1 if an observation occurred in 2017 and 0 otherwise. $Black_{im}$ took a value of 1 for Black beneficiaries and 0 otherwise. $Hispanic_{im}$ took a value of 1 for Hispanic beneficiaries and 0 otherwise. $MSA_m$was dummy variables for MSA m. $Year_t$ was a dummy variable for year t. X represented all other covariates.

β_3 measured changes in the receipt of joint replacements under CJR among White beneficiaries. We calculated $\beta_1 + \beta_3$ and $\beta_2 + \beta_3$that measured changes in the receipt of joint replacements under CJR among Black and Hispanic beneficiaries, respectively. We clustered standard errors on the MSA level to account for clustered observations within MSAs.

© 2021 Kim H et al. *JAMA Network Open.*

eFigure. The sample selection criteria

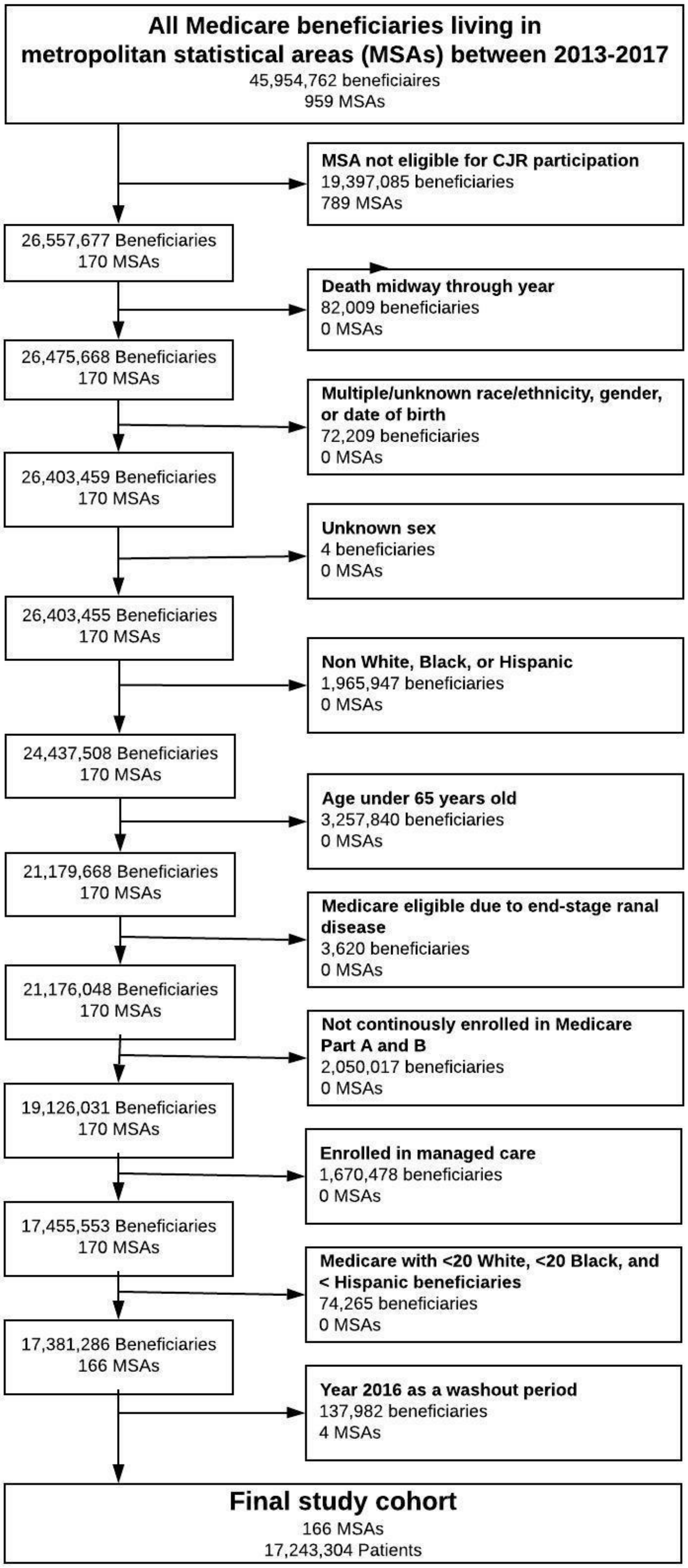

© 2021 Kim H et al. *JAMA Network Open.*

eTable 1. The parallel pre-trends assumption

	2014		2015			
	Estimate	95% CI	Estimate	95% CI	F-statistics	P value
White (N=31,834,957)	0.04	-0.3 to 0.4	0.2	-0.2 to 0.7	1.0	0.4
Black (N=3,049,671)	0.3	-0.3 to 0.8	-0.0004	-0.4 to 0.4	0.6	0.5
Hispanic (N=2,113,502)	0.1	-0.5 to 0.8	0.03	-0.7 to 0.8	0.1	0.9

Note: After limiting our sample to beneficiary-years during the pre-CJR period, we compared adjusted outcomes between treatment and control MSAs across White, Black, and Hispanic beneficiaries in year 2014 and 2015 as compared to in year 2013. If we found no differences in outcomes between treatment and control MSAs in 2014 and 2015 as compared to year 2013, we meet a parallel pre-trend assumption.

The unit of regression was each beneficiary-year in this analysis. We stratified the sample to White, Black, and Hispanic beneficiaries and ran a linear ordinary least-squares regression within each of stratified group. We regressed outcomes on an indicator of treatment MSA (vs. control), indicators of year (with year 2013 as a reference group), and interactions between an indicator of treatment MSA and indicators of year. We also adjusted for the same set of explanatory variables as those in the main analysis. As seen in the table above, we found no significant differences in the adjusted-outcome when comparing treatment and control MSAs within White, Black, and Hispanic beneficiaries in 2014 and 2015 as compared to in 2013.

© 2021 Kim H et al. *JAMA Network Open.*

eTable 2. Intention to treat analysis: Change in the rate of joint replacements per 1,000 White, Black, and Hispanic beneficiary-years under the Comprehensive Care for Joint Replacement Model (N= 51,588,590)

	Treatment MSAs			Control MSAs			Treatment vs Control		
	(1) Pre-CJR	(2) Post-CJR	(3) Difference	(4) Pre-CJR	(5) Post-CJR	(6) Difference	(7) Change associated with CJR	(8) 95% CI	(9) P-value
White	13.59	15.17	1.59	14.16	15.87	1.71	0.02	-0.37 to 0.4	0.93
Black	8.14	8.78	0.64	8.41	9.58	1.17	-0.70	-1.31 to -0.09	0.02
Hispanic	7.48	8.13	0.65	8.26	8.73	0.48	1.02	0.02 to 2.01	0.045

Note: Columns (1)-(6) show unadjusted estimates and Column (7) shows adjusted estimates.

© 2021 Kim H et al. *JAMA Network Open.*

eTable 3. Change in the rate of elective or hip fracture replacement per 1,000 White, Black, and Hispanic beneficiary-year under the Comprehensive Care for Joint Replacement Model (N=49,595,269)

	Treatment MSAs			Control MSAs			Treatment vs Control		
	(1) Pre-CJR	(2) Post-CJR	(3) Difference	(4) Pre-CJR	(5) Post-CJR	(6) Difference	(7) Change associated with CJR	(8) 95% CI	(9) P-value
White	15.95	17.32	1.36	16.61	18.13	1.52	0.04	-0.35 to 0.42	0.84
Black	8.83	9.47	0.63	9.49	10.48	0.99	-0.49	-1.13 to 0.15	0.13
Hispanic	8.7	9.26	0.56	9.49	9.91	0.42	1.03	-0.13 to 2.2	0.08

Note: Columns (1)-(6) show unadjusted estimates and Column (7) shows adjusted estimates.

© 2021 Kim H et al. *JAMA Network Open.*

eTable 4. Change in the rate of elective knee replacement per 1,000 White, Black, and Hispanic beneficiary-year under the Comprehensive Care for Joint Replacement Model (N=49,595,269)

	Treatment MSAs			Control MSAs			Treatment vs Control		
	(1) Pre-CJR	(2) Post-CJR	(3) Difference	(4) Pre-CJR	(5) Post-CJR	(6) Difference	(7) Change associated with CJR	(8) 95% CI	(9) P-value
White	8.64	9.67	1.03	9.35	10.37	1.01	0.12	-0.19 to 0.42	0.45
Black	5.43	5.74	0.31	5.91	6.72	0.81	-0.57	-1.05 to -0.08	0.02
Hispanic	5.81	6.21	0.4	6.76	6.8	0.04	0.81	0.06 to 1.57	0.04

Note: Columns (1)-(6) show unadjusted estimates and Column (7) shows adjusted estimates.

© 2021 Kim H et al. *JAMA Network Open.*

eTable 5. Change in the rate of elective hip replacement per 1,000 White, Black, and Hispanic beneficiary-year under the Comprehensive Care for Joint Replacement Model (N=49,595,269)

	Treatment MSAs			Control MSAs			Treatment vs Control		
	(1) Pre-CJR	(2) Post-CJR	(3) Difference	(4) Pre-CJR	(5) Post-CJR	(6) Difference	(7) Change associated with CJR	(8) 95% CI	(9) P-value
White	4.89	5.45	0.56	4.88	5.59	0.7	-0.08	-0.23 to 0.06	0.26
Black	2.42	2.74	0.32	2.55	2.9	0.35	-0.07	-0.33 to 0.19	0.59
Hispanic	1.6	1.88	0.28	1.63	1.94	0.31	0.27	-0.07 to 0.61	0.12

Note: Columns (1)-(6) show unadjusted estimates and Column (7) shows adjusted estimates.

© 2021 Kim H et al. *JAMA Network Open.*

References

1. Centers for Medicare & Medicaid Services. Medicare Program; Comprehensive Care for Joint Replacement Payment Model for Acute Care Hospitals Furnishing Lower Extremity Joint Replacement Services. *Fed. Regist.* 80, 73274–73554 (2015).
2. Centers for Medicare and Medicaid Services. ICD-10 Overview. https://www.cms.gov/Medicare/coding/ICD10/index.html (2018).
3. Centers for Medicare and Medicaid Services. Comprehensive Care for Joint Replacement Model. https://innovation.cms.gov/initiatives/cjr.
4. Kort, N. P., Bemelmans, Y. F. L., van der Kuy, P. H. M., Jansen, J. & Schotanus, M. G. M. Patient selection criteria for outpatient joint arthroplasty. *Knee Surg. Sports Traumatol. Arthrosc.* 25, 2668–2675 (2017).
5. Knoedler, M. A. *et al.* Risk Factors Associated With Health Care Utilization and Costs of Patients Undergoing Lower Extremity Joint Replacement. *Mayo Clin. Proc. Innov. Qual. Outcomes* 2, 248–256 (2018).
6. Courtney, P. M., Rozell, J. C., Melnic, C. M. & Lee, G.-C. Who Should Not Undergo Short Stay Hip and Knee Arthroplasty? Risk Factors Associated With Major Medical Complications Following Primary Total Joint Arthroplasty. *J. Arthroplasty* 30, 1–4 (2015).
7. Rozell, J. C., Courtney, P. M., Dattilo, J. R., Wu, C. H. & Lee, G.-C. Should All Patients Be Included in Alternative Payment Models for Primary Total Hip Arthroplasty and Total Knee Arthroplasty? *J. Arthroplasty* 31, 45–49 (2016).
8. Courtney, P. M., Boniello, A. J. & Berger, R. A. Complications Following Outpatient Total Joint Arthroplasty: An Analysis of a National Database. *J. Arthroplasty* 32, 1426–1430 (2017).
9. Pugely, A. J., Callaghan, J. J., Martin, C. T., Cram, P. & Gao, Y. Incidence of and Risk Factors for 30-Day Readmission Following Elective Primary Total Joint Arthroplasty: Analysis From the ACS-NSQIP. *J. Arthroplasty* 28, 1499–1504 (2013).
10. Cram, P. *et al.* What Drives Variation in Episode-of-care Payments for Primary TKA? An Analysis of Medicare Administrative Data. *Clin. Orthop. Relat. Res.* 473, 3337–3347 (2015).
11. Bundled Payments for Care Improvement Initiative: Archived Materials | Center for Medicare & Medicaid Innovation. https://innovation.cms.gov/initiatives/Bundled-Payments/Archived-Materials.html.

© 2021 Kim H et al. *JAMA Network Open.*
